# Distinct brain volume abnormalities in clinical high-risk individuals: pre- and post-antipsychotic treatment

**DOI:** 10.1017/S0033291726103250

**Published:** 2026-03-24

**Authors:** Wensi Zheng, Liren Zhang, Lihua Xu, Yanyan Wei, Huiru Cui, Dan Zhang, Yawen Hong, Jinyang Zhao, Siyan Liu, Tianhong Zhang, Yingying Tang, Jijun Wang

**Affiliations:** 1 Shanghai Key Laboratory of Psychotic Disorders, Shanghai Mental Health Center, Shanghai, China; 2 Department of Neurology, Changhai Hospital, Naval Medical University, Shanghai, China

**Keywords:** antipsychotics, clinical high risk for psychosis, early intervention, structural brain changes

## Abstract

Individuals at clinical high risk (CHR) for psychosis exhibit both baseline and progressive brain structural abnormalities. However, the extent to which these changes reflect neurobiological trajectories of illness progression versus iatrogenic effects of antipsychotic (AP) treatment remains unresolved. A total of 148 AP-naïve CHRs and 65 healthy controls (HCs) underwent baseline structural magnetic resonance imaging (MRI) scans. One hundred thirty CHRs received second-generation AP treatment and completed 2-month follow-up scans. HCs also completed the follow-up scans. We compared baseline and longitudinal brain volume changes between CHRs and HCs and explored the relationship between AP treatment and brain structural changes in CHR. At baseline, CHRs showed enlarged third and inferior lateral ventricles compared to HCs. Within CHRs, larger ventricular, as well as smaller hippocampus and amygdala volumes, were associated with more severe symptoms and poorer functioning. No cortical volume differences were observed between groups at baseline, nor were cortical volumes related to clinical symptoms. After 2-month AP treatment, CHRs exhibited continued ventricular enlargement, reduced accumbens volume, and widespread cortical volume loss relative to HCs. Notably, cortical volume reductions were dose-dependent, with higher AP dose correlating with more pronounced cortical reductions. Additionally, cortical volume changes were linked to treatment response, with high-dose responders showing more significant HC-referenced changes compared to high-dose non-responders, low-dose responders, and low-dose non-responders. Our findings underscore the complex, region-specific, and clinically relevant neuroanatomical changes in CHR individuals, emphasizing the critical need to account for AP exposure in CHR neuroimaging studies.

## Introduction

Accumulating evidence from magnetic resonance imaging (MRI) studies has demonstrated that individuals at clinical high risk (CHR) for psychosis already exhibit neuroanatomical abnormalities in both cortical and subcortical structures prior to the onset of full-threshold psychiatric disorders (Armio et al., 2024; Cannon et al., 2015; Collins et al., 2023; Del Re et al., 2021; Jalbrzikowski et al., 2021; Sasabayashi et al., 2020; Vissink et al., 2022; Zhao et al., 2022; Zheng et al., 2025). Although findings have varied across studies, several regions have been repeatedly implicated. Cortical abnormalities in the frontal, temporal, and cingulate have been most consistently reported, in line with their roles in cognitive control, emotion regulation, and salience processing. Structural alterations have also been observed in the parietal, occipital, and sensorimotor cortices, albeit less consistently. Regarding subcortical structures, abnormalities in the hippocampus, amygdala, thalamus, pallidum, and accumbens, as well as ventricular enlargement, are commonly reported, which collectively form cortico-striato-limbic circuits that are critical to the pathophysiology of psychosis vulnerability (Armio et al., 2024; Jiang et al., 2024; Sasabayashi et al., 2020; Seidman et al., 1999). These structural alterations are considered critical biological markers for the early detection and intervention of psychotic disorders. However, there remains an ongoing debate as to whether these abnormalities reflect primary pathological changes inherent to the illness or are secondary effects resulting from subsequent treatment interventions, particularly antipsychotic (AP) medication.

Previous studies from medication-naïve CHR populations have demonstrated discrepant anatomical alterations, including ventricular enlargement, gray matter loss, and subcortical volume changes in the absence of AP exposure, providing preliminary evidence for the hypothesis of primary pathological changes (Cannon et al., 2015; Hua et al., 2023; Jalbrzikowski et al., 2021; Sasabayashi et al., 2020; Zeng et al., 2022; Zhang, Qiu, & Lui, 2025). Some studies have even found that CHRs who subsequently convert to psychosis show more pronounced structural abnormalities compared to non-converters, suggesting a potential link between brain morphology and psychosis risk (Cannon et al., 2015; Cho et al., 2024; Collins et al., 2023). Additionally, research in non-psychotic first-degree relatives of schizophrenia patients has also revealed characteristic changes such as ventricular expansion and subcortical volume reductions compared with healthy controls (HCs), further supporting the theory that structural alterations in specific brain regions may serve as endophenotypes of genetic susceptibility to psychiatric vulnerability (Seidman et al., 1999; Staal et al., 2000).

Nevertheless, a growing body of evidence has confirmed that AP exposure can significantly influence the morphology of both cortical and subcortical brain structures. Preclinical studies in rodents and macaque monkeys have demonstrated that chronic AP administration induces distinct morphological changes (Dorph-Petersen et al., 2005; Vernon, Natesan, Modo, & Kapur, 2011) and a decrease in astrocyte and oligodendrocyte numbers (Konopaske et al., 2008). In human studies, particularly in schizophrenia populations, AP use has also been associated with gray matter loss as well as both increases and decreases in subcortical volumes (Emsley et al., 2023; Lesh et al., 2015; Liu et al., 2025; Si et al., 2024; Zeng et al., 2022; Zheng et al., 2025). Furthermore, some studies have reported a significant correlation between higher AP doses and greater gray matter reductions (Hua et al., 2023; Si et al., 2024). Our prior prospective cohort study highlighted a paradoxical yet clinically important observation: despite the ongoing controversy surrounding AP use in CHR populations (Raballo, Poletti, & Preti, 2024; Zhang et al., 2021), over 70% of CHR individuals received AP prescriptions and took APs at least 2 weeks after their first visit to the clinic (Zhang et al., 2022). This raises critical concerns about the interpretation of structural abnormalities reported in earlier CHR studies that did not adequately account for AP exposure, an omission that may have led to misattribution of medication-induced changes to disease progression itself.

Several neuroimaging studies in CHRs have attempted to explore the potential impact of AP exposure, but failed to detect significant associations (Cannon et al., 2015; Fortea et al., 2024; Jalbrzikowski et al., 2021; Sasabayashi et al., 2020). However, some studies have observed trends toward more significant structural declines associated with AP use (Cannon et al., 2015; Fortea et al., 2024; Jalbrzikowski et al., 2021). The lack of significant findings in these studies may be due to limited statistical power arising from small proportions of medicated participants and crude AP exposure assessments, which are often dichotomized as ‘on’ or ‘off’ medication at a single time point (e.g. baseline), without consideration of cumulative dose, duration, or dynamic treatment changes over time.

To address this knowledge gap, the present study employed a longitudinal design in a cohort of medication-naïve CHR individuals at the time of their initial clinical presentation. Structural MRI scans were conducted at baseline, prior to AP initiation, and again at a 2-month follow-up after AP exposure. Our goals were to: (1) characterize distinct patterns of cortical and subcortical volume changes attributable to illness progression (baseline) versus AP effects (follow-up); and (2) examine the dose-dependent and treatment-response-related effects of APs. We hypothesize that: (i) region-specific structural abnormalities related to clinical symptoms already exist at baseline in CHRs, such as previously reported changes in the ventricles, hippocampus, amygdala, and thalamus; (ii) AP exposure would induce further, distinct neuroanatomical changes, particularly in dopamine-modulated cortical–striatal regions; and (iii) the brain morphology changes induced by APs would correlate with AP dose and clinical treatment response.

## Methods

### Participants and assessments

This study was approved by the Institutional Review Board of Shanghai Mental Health Center (SMHC), and written informed consent was obtained from all participants or from parents/guardians of participants under the age of 18. A total of 148 CHR individuals were recruited from SMHC. The Structured Interview for Psychosis-Risk Syndromes (SIPS) (Miller et al., 2002) was used to determine whether participants met the criteria for CHR status. The CHR criteria consist of three subtypes: attenuated positive symptom syndrome (APSS), genetic risk and deterioration syndrome (GRDS), and brief intermittent psychotic syndrome (BIPS). These subtypes are defined based on the severity and duration of specific symptoms. The Chinese version of the SIPS, developed by our research team, has demonstrated excellent interrater reliability (intraclass correlation coefficient = 0.96, p < 0.01 for total score) and has been validated for use in Chinese populations (Zhang et al., 2014, 2021).

Inclusion criteria were as follows: (a) age between 13 and 40 years; (b) at least 6 years of formal education; (c) meeting CHR criteria based on SIPS; and (d) antipsychotic-naïve at the study entry. Exclusion criteria included: (a) a current diagnosis of any DSM-IV Axis I psychiatric disorder, as determined by the Mini-International Neuropsychiatric Interview (MINI) (Sheehan et al., 1998); (b) presence of serious medical conditions; (c) history of substance abuse or dependence; and (d) contraindications to MRI. Sixty-five HCs were also recruited using the same inclusion and exclusion criteria, with the exception that HCs did not meet criteria for a psychosis-risk syndrome.

At baseline (BL), all CHR and HC subjects underwent structural MRI scans. For CHR participants, symptom severity was assessed using the Scale of Prodromal Symptoms (SOPS), which consists of 19 items covering four symptom domains: positive, negative, disorganized, and general symptoms. In addition, global psychological, social, and occupational functioning was evaluated using the Global Assessment of Functioning (GAF) scale. The drop in GAF scores relative to 12 months prior was used to assess functional deterioration.

### Antipsychotic usage and follow-up

AP prescriptions were issued by clinicians during routine outpatient visits, and all prescribing decisions were documented in the electronic medical records system of the SMHC. This study was conducted as a naturalistic follow-up without any additional interventions or financial incentives for participants. At approximately 2 months (M2) after BL, CHRs were invited for follow-up clinical assessments and MRI scans. HCs also underwent follow-up MRI scanning at the same time point.

Only CHR individuals who had been on APs for at least 2 weeks and completed the M2 MRI scan were included in the longitudinal analysis (n = 130). It is important to note that during the early phase of AP treatment, medication type and dosage were frequently adjusted. Approximately 75% of participants had a different AP prescription at follow-up compared to their initial prescription at baseline. To accurately capture AP exposure, detailed AP use information since the BL assessment was collected at M2. This information, including AP type, daily dosage, and duration of use, was reported by the participants, confirmed by family members, and verified against clinical records. Given that AP adjustments were typically made on a weekly basis, cumulative AP exposure was quantified by multiplying the daily olanzapine-equivalent dose (Leucht et al., 2015) by the number of weeks used, providing a more accurate representation of AP impact. Based on this cumulative dose, CHR participants were classified into two groups: high-dose (HIGH, n = 57) and low-dose (LOW, n = 73), using a cutoff value of 80 (Zhang et al., 2020). To further examine whether AP-related neuroanatomical changes were purely dose-dependent or also related to treatment response, CHRs were stratified into four subgroups according to both cumulative AP dose and symptom improvement. Treatment response was defined as a ≥ 50% reduction in SOPS positive symptoms from BL to M2 (Jiang et al., 2022). Of the 130 CHRs, 122 completed clinical evaluations and were included in this analysis. The resulting subgroups were as follows: high-dose responders (HIGH_R, n = 26), high-dose non-responders (HIGH_NR, n = 27), low-dose responders (LOW_R, n = 27), and low-dose non-responders (LOW_NR, n = 42).

### MRI acquisition and processing

MRI data were acquired using a 3.0 Tesla scanner (Siemens, Verio) with a 32-channel head coil. High-resolution 3D structural images were collected using a T1-weighted MPRAGE sequence with the following parameters: repetition time (TR) = 2300 ms, echo time (TE) = 2.96 ms, field of view (FOV) = 256 × 256 mm^2^, voxel size = 1.0 × 1.0 × 1.0 mm^3^, 192 sagittal slices, slice thickness = 1 mm, and flip angle = 9°.

All MRI images at BL and M2 underwent visual inspection to identify motion artifacts and intracranial abnormalities and were preprocessed using the standard automated pipeline of FreeSurfer software (version 6.0, https://surfer.nmr.mgh.harvard.edu). The preprocessing steps included non-brain tissue removal, Talairach-like spatial normalization, gray/white matter segmentation, intensity normalization, gray/white boundary tessellation, topology correction, and surface deformation. After preprocessing, each dataset was visually reviewed, and all identified errors were manually corrected and rechecked. For longitudinal analyses, we used FreeSurfer’s longitudinal processing stream, which creates an unbiased within-subject template from all time-point scans of each participant and then registers each time-point scan to this template. This approach increases signal-to-noise ratio and improves sensitivity for detecting subtle brain changes over time (Reuter, Schmansky, Rosas, & Fischl, 2012).

Considering the widespread but inconsistent reports of structural abnormalities in previous CHR studies, this research employs a whole-brain region-of-interest (ROI) analysis. Eight cortical ROIs were defined using the Desikan–Killiany atlas (Desikan et al., 2006), including the orbitofrontal (OFC), lateral prefrontal (LPFC), medial prefrontal (MPFC), lateral temporal (LTC), medial temporal (MTC), somatomotor (SMC), parietal (PC), and occipital cortex (OCC) (Cho et al., 2024). The corresponding Desikan–Killiany labels for each region are provided in Supplementary Table S1. In addition, three classic ventricular regions (third ventricle, lateral ventricle, and inferior lateral ventricle) and seven subcortical structures commonly explored in neuroimaging research (thalamus, caudate, putamen, pallidum, hippocampus, amygdala, and accumbens) were also included (Fischl et al., 2002). Volumetric data from all ROIs, as well as intracranial volume (ICV), were extracted for further statistical analysis.

### Statistical analyses

All statistical analyses were performed using R software (version 4.3.2). For group comparisons of demographic variables, independent-samples t-tests or Mann–Whitney U test were used for continuous variables, depending on whether it meets the normality assumption. Chi-square tests were applied for categorical variables.

To examine baseline differences between CHR and HC groups, general linear models were conducted for each ROI. In addition, partial correlation analyses were performed to assess the association between baseline clinical scale scores and ROI volumes. All group comparisons and correlation analyses were conducted while controlling for sex, age, and ICV as covariates, and all resulting p-values were corrected for multiple comparisons using the false discovery rate (FDR) method.

Linear mixed-effects (LME) models were fitted using the lme4 package in R to examine differences between CHR and HC groups from BL to M2 across all ROIs, with sex, age, ICV, and scan interval included as covariates. The p-values for the Group*Time interaction were FDR corrected for whole-brain, and ROIs with an FDR-corrected p < 0.05 were considered to exhibit a significant divergence in longitudinal trajectory and were carried forward for further analysis. In the identified ROIs, simple-effect analyses using the emmeans package were conducted to compare BL versus M2 within the CHR and HC groups, resulting in p-values from all pairwise comparisons (two comparisons per ROI) were pooled and FDR-corrected. In addition, the partial η^2^ (with 95% confidence intervals) of the interaction effects was calculated in these ROIs as well to quantify the effect size.

To investigate whether the brain changes observed in CHR participants were related to AP exposure, we performed partial correlation analyses between cumulative AP dose and the volume change rate ([M2-BL]/BL) for each of the ROIs identified, controlling for sex, age, ICV, scan interval, and baseline SOPS total score. The resulting p-values were FDR-corrected across all tested ROIs. Furthermore, as an additional exploratory step, LME models were also constructed to assess the effect of AP dosage (HIGH versus LOW versus HC) and the combined effects of dosage and treatment response (HIGH_R versus HIGH_NR versus LOW_R versus LOW_NR versus HC) with sex, age, ICV, and scan interval as covariates. In these models, FDR correction was also applied across the tested ROIs for the interaction terms to control Type I error.

## Results

### Sample characteristics

Demographic and clinical characteristics are displayed in Table 1. At BL, there were no significant differences in sex, age, or education between the CHR and HC groups (all p > 0.05). Also, no significant differences were observed in sex, age, education, SOPS total score, SOPS domain score, GAF current score, or GAF dropout rate over the past year between the LOW and HIGH subgroups (all p > 0.05). However, the HIGH group had a significantly higher AP dose (z = 9.77, p < 0.001) and longer scan interval (z = 2.30, p = 0.021) compared to the LOW group. Therefore, the scan interval was included as a covariate in all longitudinal analyses, as described in the Methods section.

**Table 1.** Socio-demographic and clinical characteristics of the sample

	HC (*n* = 65)	CHR (*n* = 148)	HC versus Whole CHR		AP dose (*n* = 130)		LOW versus HIGH	
			T /Z /χ2	*p*	LOW (*n* = 73)	HIGH (*n* = 57)	T /Z /χ2	*p*
Age	17 (16, 22)	17 (15, 21)	1.93	0.053	17 (16, 21)	16 (15, 21)	1.14	0.255
Sex			1.61	0.204			0.31	0.577
Female, *n* (%)	29 (44.6%)	80 (54.1%)			42 (57.5%)	30 (52.6%)		
Male, *n* (%)	36 (55.4%)	68 (45.9%)			31 (42.5%)	27 (47.4%)		
Education	11 (9, 13)	10 (9, 13)	1.94	0.053	10 (9, 13)	9 (8, 12)	1.67	0.096
SOPS total	/	38.07 (11.53)	/	/	39.01 (10.88)	39.47 (10.97)	0.24	0.812
SOPS positive	/	10 (8, 13)	/	/	11 (8, 13)	10 (8, 12)	0.97	0.330
SOPS negative	/	12.98 (5.97)	/	/	12.40 (5.58)	13.72 (6.40)	−1.24	0.219
SOPS disorganized	/	6 (4, 8.75)	/	/	6 (4, 9)	7 (4, 8)	−0.04	0.974
SOPS general	/	9.15 (2.87)	/	/	9.36 (2.83)	8.88 (2.92)	0.94	0.349
GAF current	/	56 (51, 60)	/	/	55 (51, 60)	56 (50, 59)	0.91	0.363
GAF drop rate	/	0.30 (0.25, 0.35)	/	/	0.30 (0.25, 0.34)	0.30 (0.26, 0.36)	1.09	0.276
Scan interval	/	9 (8, 12)	/	/	9 (8, 11)	10 (9, 12)	2.30	0.021*
AP dose	/	80 (42, 110)	/	/	45 (30, 60)	120 (100, 158)	9.77	<0.001**

*Note:* Values are mean (SD) or median (P25, P75) as appropriate. HC, healthy controls; CHR, clinical high-risk for psychosis; LOW, low-dose-antipsychotic group; HIGH, high-dose-antipsychotic group; AP, antipsychotic; SOPS, scale of prodromal symptoms total scores; GAF, global assessment of functioning scores. *Significant at *p* < 0.05, **Significant at *p* < 0.01, and ***Significant at *p* < 0.001.

### Baseline group differences between CHR and HC, and correlations between clinical symptoms and ROI volumes in CHRs

**Figure 1.**
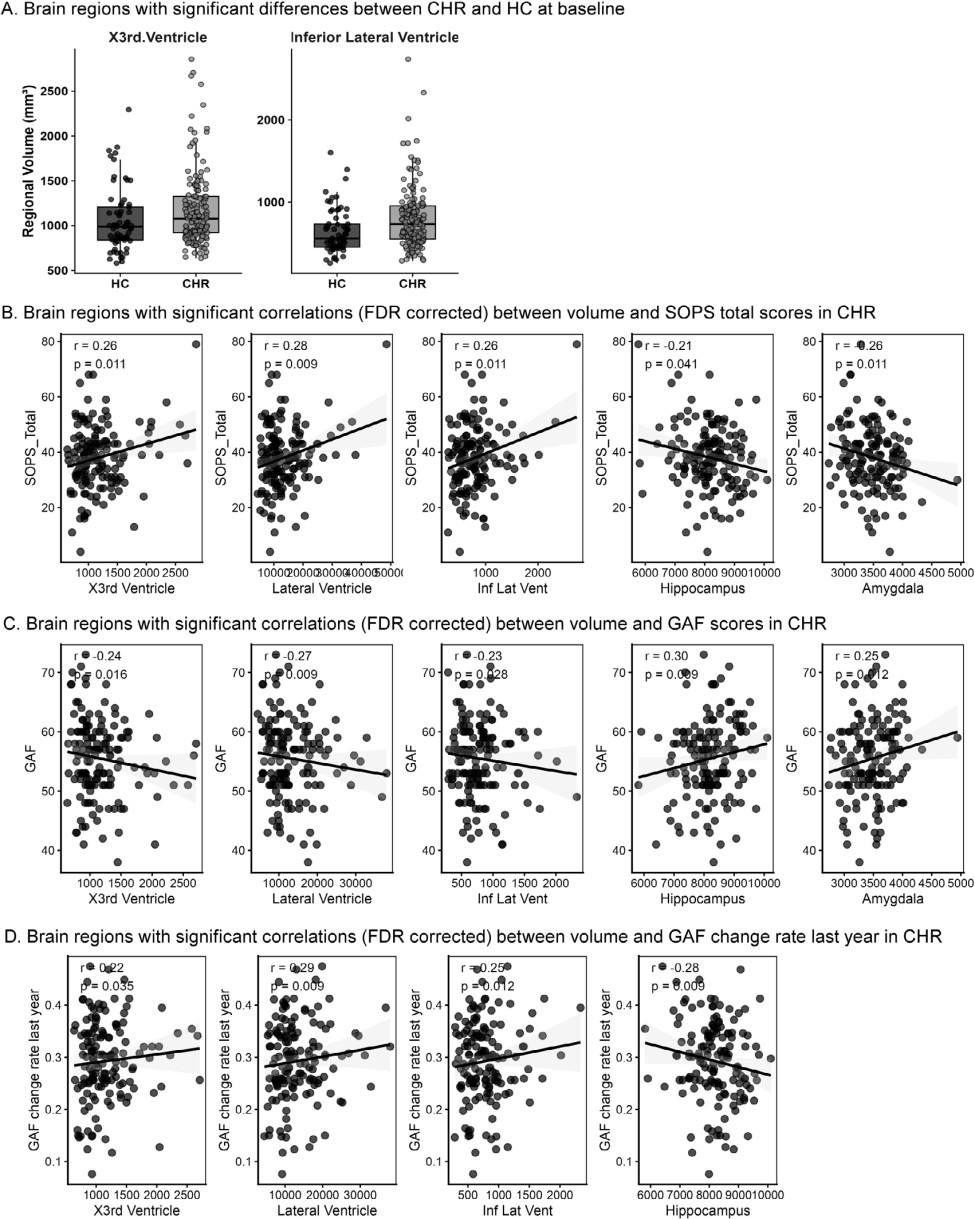
Baseline group differences and correlations between clinical symptoms and brain volumes in CHRs. CHR participants had significantly larger volumes in the third and inferior lateral ventricles compared to HCs (a). In the CHR group, a higher SOPS total score was positively correlated with ventricular volumes and negatively correlated with hippocampal and amygdala volumes (b). A higher GAF score was negatively correlated with ventricular volumes and positively correlated with hippocampal and amygdala volumes (c). A greater GAF drop rate was also associated with larger ventricular volumes and smaller hippocampal volume (d). All results controlled for sex, age, and ICV as covariates and were FDR corrected.

At BL, CHR participants showed significantly larger volumes in the third ventricle (β[SE] = 164.68[52.39], FDR-p = 0.017) and inferior lateral ventricle (β[SE] = 181.55[51.72], FDR-p = 0.010) compared to HC (Figure 1a, Supplementary Table S2). Importantly, within the CHR group, greater symptom severity, poorer current functioning, and greater functional decline over the past year were associated with enlarged ventricular volumes and reduced hippocampus and amygdala volumes (Figure 1b–d, Supplementary Table S3). Specifically, volumes of the third, lateral, and inferior lateral ventricle were positively correlated with SOPS total scores (r = 0.26, FDR-p = 0.011; r = 0.28, FDR-p = 0.009; r = 0.26, FDR-p = 0.011, respectively) and the rate of GAF decline over the past year (r = 0.22, FDR-p = 0.035; r = 0.29, FDR-p = 0.009; r = 0.25, FDR-p = 0.012, respectively), and negatively correlated with current GAF scores (r = −0.24, FDR-p = 0.016; r = −0.27, FDR-p = 0.009; r = −0.23, FDR-p = 0.028, respectively). In contrast, hippocampal volume showed negative correlations with SOPS total scores (r = −0.21, FDR-p = 0.041) and GAF decline rates (r = −0.28, FDR-p = 0.009), and a positive correlation with current GAF scores (r = 0.30, FDR-p = 0.009). Amygdala volume was negatively associated with SOPS total scores (r = −0.26, FDR-p = 0.011) and positively associated with current GAF scores (r = 0.25, FDR-p = 0.012). All cortical ROI volumes did not differ between CHR and HC, nor did they correlate with symptoms of CHR.

### Longitudinal neuroanatomical changes in CHR versus HC groups

**Figure 2.**
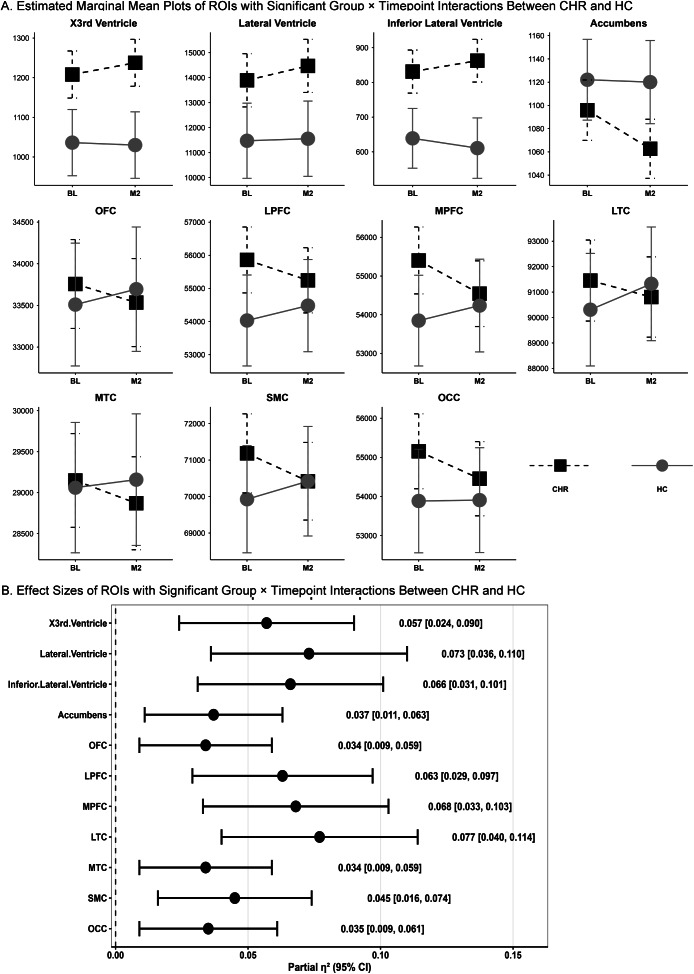
Longitudinal brain volume change differences between CHRs and HCs. CHRs exhibited significantly greater third, lateral, and inferior lateral ventricular enlargement and more reductions in the volumes of the accumbens, orbitofrontal (OFC), lateral prefrontal (LPFC), medial prefrontal (MPFC), lateral temporal (LTC), medial temporal (MTC), somatomotor (SMC), and occipital (OCC) cortex during the follow-up period compared to HCs (FDR corrected) (a). The linear mixed-effects model showed small-to-moderate effect sizes for the time-by-group interaction (b).

LME models revealed significant group*time interactions in multiple brain regions, indicating divergent longitudinal trajectories of brain volume between CHR and HC individuals (Figure 2, Table 2). Specifically, compared to HCs, CHRs showed significantly greater increases over time in the volumes of the third (β[SE] = 33.75[10.37], FDR-p = 0.002, partial η^2^ = 0.057), lateral (β[SE] = 494.26[126.41], FDR-p = 0.001, partial η^2^ = 0.073), and inferior lateral ventricle (β[SE] = 59.54[16.07], FDR-p = 0.001, partial η^2^ = 0.066). Conversely, significant reductions were observed in the accumbens (β[SE] = −31.20[11.45], FDR-p = 0.016, partial η^2^ = 0.037) and widespread cortical ROIs, including OFC (β[SE] = −407.22[156.03], FDR-p = 0.016, partial η^2^ = 0.034), LPFC (β[SE] = −1063.24[294.34], FDR-p = 0.002, partial η^2^ = 0.063), MPFC (β[SE] = −1248.76[331.13], FDR-p = 0.002, partial η^2^ = 0.068), LTC (β[SE] = −1664.08[413.48], FDR-p = 0.002, partial η^2^ = 0.077), MTC (β[SE] = −375.51 [143.90], FDR-p = 0.016, partial η^2^ = 0.034), SMC (β[SE] = −1259.37[414.67], FDR-p = 0.007, partial η^2^ = 0.045), and OCC (β[SE] = −723.33[270.87], FDR-p = 0.008, partial η^2^ = 0.035).

**Table 2.** Results of linear mixed-effect models and correlations for all ROIs

	Time (BL-M2) * group (CHR-HC) Interaction Effects			Simple effects: HC (BL-M2)			Simple effects: CHR (BL-M2)			Correlations with AP dose		
	β (SE)	*p*	FDR-*p*	β (SE)	*p*	FDR-*p*	β (SE)	*p*	FDR-*p*	r	*p*	FDR-*p*
X3rd ventricle	35.75 (10.37)	<0.001	0.002**	6.25 (15.35)	0.684	0.753	−29.50 (12.49)	0.019	0.140	0.18	0.041	0.075
Lateral ventricle	494.26 (126.41)	<0.001	0.001**	−81.74 (187.36)	0.663	0.753	−576.00 (152.47)	<0.001	0.005**	0.16	0.071	0.112
Inferior lateral ventricle	59.54 (16.07)	<0.001	0.001**	28.17 (23.67)	0.235	0.398	−31.37 (19.25)	0.105	0.266	0.023	0.803	0.803
Thalamus	−31.67 (48.59)	0.515	0.580	/	/	/	/	/	/	/	/	/
Caudate	−8.09 (24.70)	0.744	0.744	/	/	/	/	/	/	/	/	/
Putamen	79.84 (47.11)	0.092	0.110	/	/	/	/	/	/	/	/	/
Pallidum	40.89 (22.20)	0.067	0.086	/	/	/	/	/	/	/	/	/
Hippocampus	−70.51 (35.44)	0.048	0.067	/	/	/	/	/	/	/	/	/
Amygdala	−7.71 (21.26)	0.717	0.744	/	/	/	/	/	/	/	/	/
Accumbens	−31.20 (11.45)	0.007	0.016*	2.01 (16.55)	0.904	0.947	33.20 (13.43)	0.014	0.140	−0.07	0.432	0.475
OFC	−407.22 (156.03)	0.010	0.016*	−185.27 (229.30)	0.420	0.544	221.96 (186.42)	0.235	0.398	−0.30	<0.001	0.007**
LPFC	−1063.24 (294.34)	<0.001	0.002**	−448.35 (432.47)	0.301	0.473	614.88 (351.59)	0.082	0.266	−0.21	0.018	0.040*
MPFC	−1248.76 (331.13)	<0.001	0.002**	−390.78 (482.43)	0.419	0.544	857.98 (391.83)	0.030	0.140	−0.13	0.142	0.195
LTC	−1664.08 (413.48)	<0.001	0.002**	−1018.42 (609.08)	0.096	0.266	645.66 (495.31)	0.194	0.388	−0.26	0.004	0.020*
MTC	−375.51 (143.90)	0.010	0.016*	−97.76 (212.07)	0.645	0.753	277.74 (172.47)	0.109	0.266	−0.24	0.008	0.028*
SMC	−1259.37 (414.67)	0.003	0.007**	−496.50 (604.13)	0.412	0.544	762.87 (490.68)	0.121	0.267	−0.10	0.290	0.355
PC	−1291.12 (591.24)	0.041	0.061	/	/	/	/	/	/	/	/	/
OCC	−723.33 (270.87)	0.008	0.016*	−23.13 (398.37)	0.954	0.954	700.20 (323.90)	0.032	0.140	−0.22	0.016	0.040*

*Note:* BL, baseline; M2, month 2; CHR, clinical high risk for psychosis; HC, health control; β, estimated effect; SE, Standard Error; FDR, false discovery rate; OFC, orbitofrontal cortex; LPFC, lateral prefrontal cortex; MPFC, medial prefrontal cortex; LTC, lateral temporal cortex; MTC, medial temporal cortex; SMC, somatomotor cortex; PC, parietal cortex; OCC, occipital cortex. *Significant at *p* < 0.05, **Significant at *p* < 0.01, and ***Significant at *p* < 0.001.

Simple-effect analyses showed that significant interaction effects were primarily driven by volume changes in the CHR group in the third ventricle (β[SE] = 29.50[12.49], p = 0.019, FDR-p = 0.140), lateral ventricles (β[SE] = 576.00[152.47], p < 0.001, FDR-p = 0.005), accumbens (β[SE] = −33.20[13.43], p = 0.014, FDR-p = 0.140), MPFC (β[SE] = −857.98[391.83], p = 0.030, FDR-p = 0.140), and OCC (β[SE] = −700.20[323.90], p = 0.032, FDR-p = 0.140). In contrast, HC individuals showed no significant volume changes over time in all regions (all p and FDR-p > 0.05) (Figure 2, Table 2).

### Correlations between AP dose and brain volume change rates in CHRs

**Figure 3.**
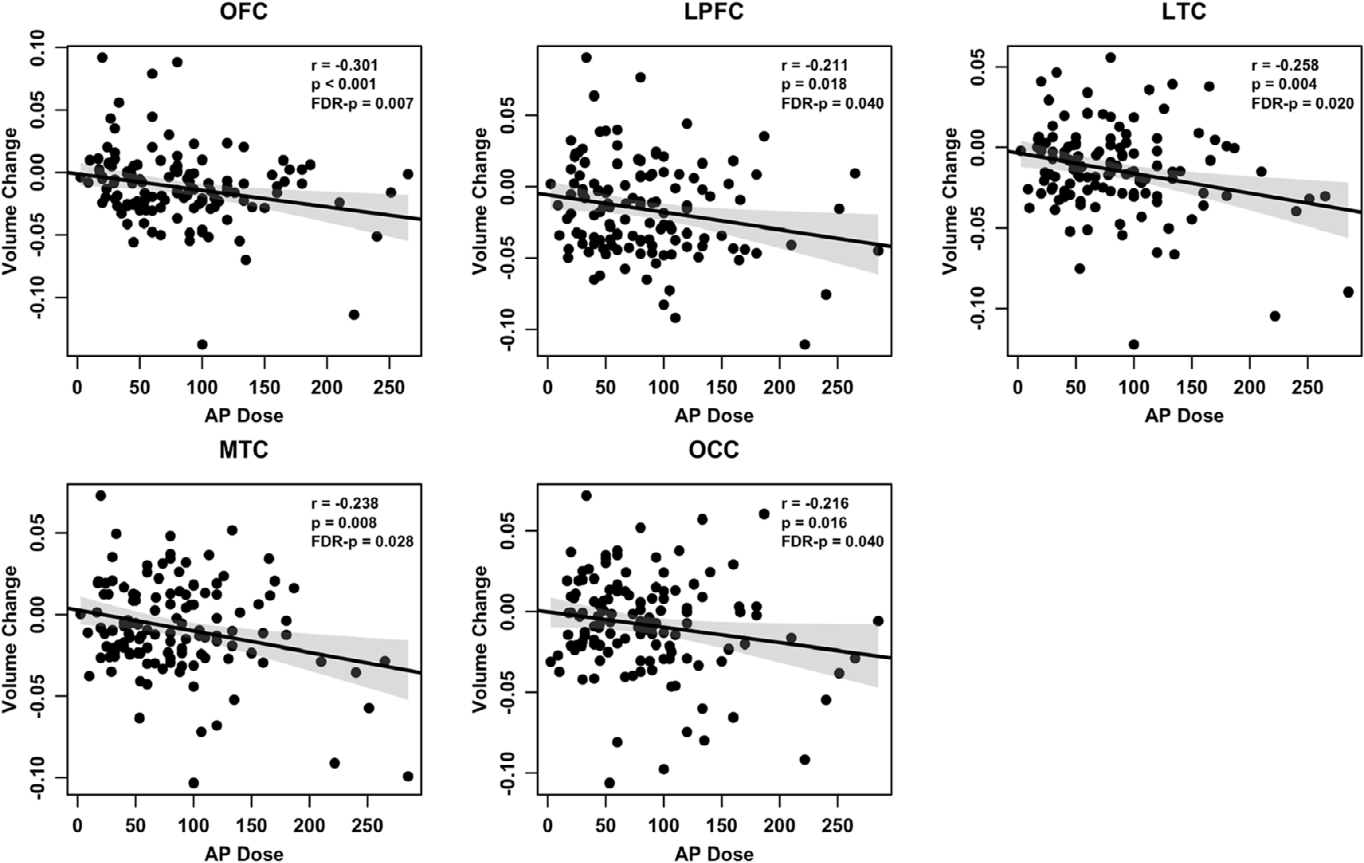
Correlations between cumulative antipsychotic dose and longitudinal brain volume changes in CHRs. Cumulative antipsychotic dose was significantly negatively correlated with the rate of cortical volume changes in several brain regions, including the orbitofrontal (OFC), lateral prefrontal (LPFC), lateral temporal (LTC), medial temporal (MTC), and occipital (OCC) cortex. This analysis controlled for sex, age, ICV, scan interval, and baseline SOPS total score, with FDR correction applied.

Among the 11 ROIs showing significant longitudinal CHR versus HC differences, significant negative correlations between AP doses and brain volume change rates were observed in several regions within CHR group, including the OFC (r = −0.30, FDR-p = 0.007), LPFC (r = −0.21, FDR-p = 0.040), LTC (r = −0.26, FDR-p = 0.020), MTC (r = −0.24, FDR-p = 0.028), and OCC (r = −0.22, FDR-p = 0.040), after controlling for sex, age, ICV, scan interval, and baseline SOPS total score (Table 2, Figure 3). These findings suggest that higher cumulative AP exposure is associated with more pronounced volume reduction in these brain regions.

### Dose- and response-related effects of AP on longitudinal brain volume changes

Exploratory longitudinal subgroup analysis of the 11 ROIs revealed significant group*time interactions, while distinct patterns emerged between ventricular/subcortical and cortical regions (Supplementary Figure S1, Supplementary Table S4). In the ventricular regions (third, lateral, and inferior lateral ventricles) and the accumbens, both the LOW and HIGH dosage groups showed more significant volume changes compared to HCs. In contrast, cortical regions (especially OFC, LPFC, MPFC, LTC, MTC, and OCC) exhibited a dose-related pattern: the HIGH group showed more pronounced reductions, while the LOW group showed milder or no significant changes. Further subgroup analyses (LOW_R versus LOW_NR versus HIGH_R versus HIGH_NR versus HC) revealed similar findings. Compared to HCs, changes in the ventricles and accumbens were observed across all subgroups. However, significant changes in cortical ROIs (LPFC, MPFC, LTC, MTC, SMC, and OCC) were most pronounced in the HIGH_R group, with minimal or no changes in other subgroups. These results suggest that the reductions in cortical volumes following AP exposure are not only dose-related but may also be associated with treatment response.

## Discussion

To our knowledge, this is the first longitudinal study to investigate both pre- and post-AP exposure structural brain changes in CHR individuals. We identified both baseline neuroanatomical abnormalities and progressive changes following initial AP treatment and further demonstrated that these alterations are modulated by APs in a dose-dependent manner and may be associated with treatment response.

Medication-naïve CHR individuals exhibited baseline third and inferior lateral ventricular enlargement versus HCs, with greater ventricular and smaller hippocampus/amygdala volumes correlating with worse symptoms/function. These findings suggest that ventricular enlargement and hippocampus/amygdala volume reductions may represent primary structural manifestations of psychosis vulnerability independent of treatment effects. This interpretation is supported by previous evidence: numerous studies have shown that volume reductions in subcortical regions, especially the hippocampus and amygdala, are hallmark neurobiological features of psychotic spectrum disorders and can be observed across disease stages, including in drug-naïve patients and CHR individuals (Jalbrzikowski et al., 2021; Nelson et al., 2025; Vissink et al., 2022; Zeng et al., 2022). A large-scale, data-driven study using global multi-site data has further identified early subcortical-predominant loss of the hippocampus and amygdala as potential neuroanatomical origins of psychotic disorders (Jiang et al., 2024). Importantly, studies in first-degree relatives of schizophrenia patients have also revealed ventricular enlargement and volume reductions in the hippocampus–amygdala complex, supporting the notion that these alterations may reflect heritable endophenotypes of psychotic disorders (Seidman et al., 1999; Staal et al., 2000).

As a key hub of the limbic system, the hippocampus is crucial for contextual memory, declarative memory, and the regulation of emotional information (Knierim, 2015), while the amygdala is central to emotional processing, particularly fear and threat evaluation (Kirstein, Güntürkün, & Ocklenburg, 2023). Volume reductions in these regions may be closely related to cognitive and emotional dysfunctions in CHR individuals. For instance, hippocampal dysfunction may impair the encoding and retrieval of contextual memories, which is related to difficulties in orientation and memory in real-world settings, while abnormal amygdala function may contribute to emotional dysregulation, deficits in social cognition, and the emergence of negative symptoms. Ventricular enlargement, typically interpreted as reflecting increased cerebrospinal fluid pressure and reduced overall volumes of surrounding brain parenchyma (Nakadate & Kamata, 2022), could serve as an early sensitive indicator of compromised overall brain structural integrity, closely related to disease risk and severity. During the CHR phase, these brain abnormalities may be related to pathological processes such as neurodevelopmental alterations, abnormal synaptic pruning, oligodendrocyte dysfunction, and neuroinflammation (Nakadate & Kamata, 2022; Sinnecker et al., 2020), gradually leading to the onset of early symptoms.

Following 2-month AP exposure, we observed continued ventricular enlargement, reductions in the volume of the accumbens, and widespread cortical volume loss in CHR individuals. While it is not possible to completely rule out the influence of illness progression during this period, we believe that the observed brain changes are primarily AP-driven. First, most CHR participants had prolonged untreated symptoms for several months without baseline cortical differences, yet developed significant cortical reductions within just 2 months of treatment, a timeframe more consistent with medication effects than natural illness progression. Second, cortical changes showed clear dose–response relationships with APs, further supporting a medication-mediated mechanism. Third, prior research in schizophrenia has also suggested that illness duration alone may have a limited impact on brain structural features when compared to AP exposure, reporting substantial differences in brain structure between drug-naïve and AP-treated first-episode patients, whereas relatively modest differences were observed between drug-naïve first-episode and untreated chronic patients (Zeng et al., 2022).

Multiple cross-sectional and longitudinal studies in schizophrenia have reported more extensive and widespread gray matter loss after AP treatment (Hua et al., 2023; Jiang et al., 2022; Lesh et al., 2015; Si et al., 2024; Zeng et al., 2022). Some studies have also found a significant correlation between higher AP doses and greater gray matter reductions (Hua et al., 2023; Si et al., 2024). We observed a similar phenomenon in CHR individuals, where higher AP doses were associated with more pronounced volume decreases in widespread cortical areas. Although the mechanisms by which APs affect brain structure remain unclear, neuroinflammatory models offer a potential explanation. Growing evidence implicates neuroinflammation in the pathophysiology of schizophrenia, and AP treatment has been associated with anti-inflammatory effects, which may reduce extracellular volume and activated glial cells (Lesh et al., 2015; Tourjman et al., 2013). Higher AP doses may exert stronger anti-inflammatory modulation, leading to greater volume reduction. Another possible mechanism involves AP-related neurotoxicity, where higher doses may lead to more loss of glial cells or reduced pyramidal cell synaptic density, contributing to volume decline.

Interestingly, we found that high-dose responders showed the most cortical volume reductions, suggesting that such decreases might be related to the therapeutic effects of AP. This pattern aligns with a prior longitudinal study in schizophrenia, which reported that responders showed more significant cortical thinning after 3 months of AP treatment but exhibited stronger cortico-cortical covariance and greater network integration (Jiang et al., 2022). Another study similarly found that AP-medicated schizophrenia patients exhibited thinner frontotemporal cortices, yet showed superior dorsolateral prefrontal cortex activation and better task performance compared to unmedicated peers (Lesh et al., 2015). Together with our findings, these results challenge the simplistic view that AP-associated gray matter reduction reflects mere neurotoxicity. Instead, it may relate to reduced neuroinflammation, modulation of dopaminergic and glutamatergic systems, and enhanced cortical covariance and network integration, thereby contributing to symptomatic improvement.

In contrast, the observed post-AP ventricular enlargement and accumbens volume reduction lack a dose–response relationship. Ventricular enlargement is typically associated with increased cerebrospinal fluid pressure and overall brain volume loss (including the cortex) (Nakadate & Kamata, 2022; Sinnecker et al., 2020), which may be sensitive even at low doses. Accumbens volume reduction may be more closely tied to long-term changes in emotional and reward system function (Zhao et al., 2025), which could be influenced more by neurodevelopmental abnormalities or ongoing disease progression than by AP exposure. Therefore, while medication affects ventricular and accumbens volumes, these changes do not show the same clear dose–response relationship as cortical regions.

Our study highlights distinct patterns of neuroanatomical changes in CHR individuals before and after AP treatment. Hippocampus and amygdala abnormalities may represent early markers of disease vulnerability, offering potential for identifying high-risk individuals and guiding early intervention strategies. In contrast, cortical changes following AP use may be more informative for predicting treatment response and evaluating therapeutic outcomes. These findings underscore the need to interpret structural brain changes within a stage-specific framework, rather than viewing them as uniform, and support a more nuanced understanding of brain alterations across the psychosis spectrum.

Several limitations should be acknowledged. First, the absence of a medication-free CHR control group limits our ability to fully disentangle illness-related from treatment-related effects. Second, given that structural changes typically evolve slowly, our 2-month follow-up may capture only early treatment effects without reflecting longer term trajectories. Third, all participants were recruited from a single center (SMHC) and consisted entirely of Chinese individuals, which may limit the generalizability of our findings. Future research should include longer term longitudinal designs to track neuroanatomical trajectories and explore the molecular and cellular mechanisms underlying the observed changes, which could help establish neuroimaging biomarkers to guide personalized intervention strategies in CHR.

In conclusion, our findings underscore the complexity, regional specificity, and clinical relevance of neuroanatomical changes in CHR individuals, which highlight the importance of accounting for AP exposure in CHR neuroimaging studies. The divergent structural trajectories in specific regions may offer valuable insights into the biological underpinnings of psychosis risk and guide the development of personalized therapeutic strategies.

## Supporting information

10.1017/S0033291726103250.sm001 Zheng et al. supplementary material Zheng et al. supplementary material

## Data Availability

Due to the ethical approval conditions, the data supporting this study cannot be made openly available.

## Supplementary material

The supplementary material for this article can be found at http://doi.org/10.1017/S0033291726103250.

## References

[r1] Armio, R. L., Laurikainen, H., Ilonen, T., Walta, M., Sormunen, E., Tolvanen, A., … Hietala, J. (2024). Longitudinal study on hippocampal subfields and glucose metabolism in early psychosis. Schizophrenia (Heidelb), 10(1), 66. 10.1038/s41537-024-00475-z. 39085221 PMC11291638

[r2] Cannon, T. D., Chung, Y., He, G., Sun, D., Jacobson, A., van Erp, T. G., … Heinssen, R. (2015). Progressive reduction in cortical thickness as psychosis develops: A multisite longitudinal neuroimaging study of youth at elevated clinical risk. Biological Psychiatry, 77(2), 147–157. 10.1016/j.biopsych.2014.05.023. 25034946 PMC4264996

[r3] Cho, K. I. K., Zhang, F., Penzel, N., Seitz-Holland, J., Tang, Y., Zhang, T., … Pasternak, O. (2024). Excessive interstitial free-water in cortical gray matter preceding accelerated volume changes in individuals at clinical high risk for psychosis. Molecular Psychiatry, 29(11), 3623–3634. 10.1038/s41380-024-02597-3. 38830974

[r4] Collins, M. A., Ji, J. L., Chung, Y., Lympus, C. A., Afriyie-Agyemang, Y., Addington, J. M., … Cannon, T. D. (2023). Accelerated cortical thinning precedes and predicts conversion to psychosis: The NAPLS3 longitudinal study of youth at clinical high-risk. Molecular Psychiatry, 28(3), 1182–1189. 10.1038/s41380-022-01870-7. 36434057 PMC10005940

[r5] Del Re, E. C., Stone, W. S., Bouix, S., Seitz, J., Zeng, V., Guliano, A., … Niznikiewicz, M. A. (2021). Baseline cortical thickness reductions in clinical high risk for psychosis: Brain regions associated with conversion to psychosis versus non-conversion as assessed at one-year follow-up in the Shanghai-at-risk-for-psychosis (SHARP) study. Schizophrenia Bulletin, 47(2), 562–574. 10.1093/schbul/sbaa127. 32926141 PMC8480195

[r6] Desikan, R. S., Ségonne, F., Fischl, B., Quinn, B. T., Dickerson, B. C., Blacker, D., … Killiany, R. J. (2006). An automated labeling system for subdividing the human cerebral cortex on MRI scans into gyral based regions of interest. NeuroImage, 31(3), 968–980. 10.1016/j.neuroimage.2006.01.021. 16530430

[r7] Dorph-Petersen, K. A., Pierri, J. N., Perel, J. M., Sun, Z., Sampson, A. R., & Lewis, D. A. (2005). The influence of chronic exposure to antipsychotic medications on brain size before and after tissue fixation: A comparison of haloperidol and olanzapine in macaque monkeys. Neuropsychopharmacology, 30(9), 1649–1661. 10.1038/sj.npp.1300710. 15756305

[r8] Emsley, R., du Plessis, S., Phahladira, L., Luckhoff, H. K., Scheffler, F., Kilian, S., … Asmal, L. (2023). Antipsychotic treatment effects and structural MRI brain changes in schizophrenia. Psychological Medicine, 53(5), 2050–2059. 10.1017/s0033291721003809. 35441587 PMC10106303

[r9] Fischl, B., Salat, D. H., Busa, E., Albert, M., Dieterich, M., Haselgrove, C., … Dale, A. M. (2002). Whole brain segmentation: Automated labeling of neuroanatomical structures in the human brain. Neuron, 33(3), 341–355. 10.1016/s0896-6273(02)00569-x. 11832223

[r10] Fortea, A., van Eijndhoven, P., Calvet-Mirabent, A., Ilzarbe, D., Batalla, A., de la Serna, E., … Sugranyes, G. (2024). Age-related change in cortical thickness in adolescents at clinical high risk for psychosis: A longitudinal study. European Child & Adolescent Psychiatry, 33(6), 1837–1846. 10.1007/s00787-023-02278-6. 37644217

[r11] Hua, J. P. Y., Loewy, R. L., Stuart, B., Fryer, S. L., Niendam, T. A., Carter, C. S., … Mathalon, D. H. (2023). Cortical and subcortical brain morphometry abnormalities in youth at clinical high-risk for psychosis and individuals with early illness schizophrenia. Psychiatry Research. Neuroimaging, 332, 111653. 10.1016/j.pscychresns.2023.111653. 37121090 PMC10362971

[r12] Jalbrzikowski, M., Hayes, R. A., Wood, S. J., Nordholm, D., Zhou, J. H., Fusar-Poli, P., … Hernaus, D. (2021). Association of Structural Magnetic Resonance Imaging Measures with Psychosis Onset in individuals at clinical high risk for developing psychosis: An ENIGMA working group mega-analysis. JAMA Psychiatry, 78(7), 753–766. 10.1001/jamapsychiatry.2021.0638. 33950164 PMC8100913

[r13] Jiang, Y., Luo, C., Wang, J., Palaniyappan, L., Chang, X., Xiang, S., … Feng, J. (2024). Neurostructural subgroup in 4291 individuals with schizophrenia identified using the subtype and stage inference algorithm. Nature Communications, 15(1), 5996. 10.1038/s41467-024-50267-3. PMC11252381 39013848

[r14] Jiang, Y., Wang, Y., Huang, H., He, H., Tang, Y., Su, W., … Luo, C. (2022). Antipsychotics effects on network-level reconfiguration of cortical morphometry in first-episode schizophrenia. Schizophrenia Bulletin, 48(1), 231–240. 10.1093/schbul/sbab082. 34313782 PMC8781340

[r15] Kirstein, C. F., Güntürkün, O., & Ocklenburg, S. (2023). Ultra-high field imaging of the amygdala - a narrative review. Neuroscience and Biobehavioral Reviews, 152, 105245. 10.1016/j.neubiorev.2023.105245. 37230235

[r16] Knierim, J. J. (2015). The hippocampus. Current Biology: CB, 25(23), R1116–R1121. 10.1016/j.cub.2015.10.049. 26654366

[r17] Konopaske, G. T., Dorph-Petersen, K. A., Sweet, R. A., Pierri, J. N., Zhang, W., Sampson, A. R., & Lewis, D. A. (2008). Effect of chronic antipsychotic exposure on astrocyte and oligodendrocyte numbers in macaque monkeys. Biological Psychiatry, 63(8), 759–765. 10.1016/j.biopsych.2007.08.018. 17945195 PMC2386415

[r18] Lesh, T. A., Tanase, C., Geib, B. R., Niendam, T. A., Yoon, J. H., Minzenberg, M. J., … Carter, C. S. (2015). A multimodal analysis of antipsychotic effects on brain structure and function in first-episode schizophrenia. JAMA Psychiatry, 72(3), 226–234. 10.1001/jamapsychiatry.2014.2178. 25588194 PMC4794273

[r19] Leucht, S., Samara, M., Heres, S., Patel, M. X., Furukawa, T., Cipriani, A., … Davis, J. M. (2015). Dose equivalents for second-generation antipsychotic drugs: The classical mean dose method. Schizophrenia Bulletin, 41(6), 1397–1402. 10.1093/schbul/sbv037. 25841041 PMC4601707

[r20] Liu, Z., Lu, W., Zou, W., Gao, Y., Li, X., Xu, G., … Shao, R. (2025). A preliminary study of brain developmental features of bipolar disorder familial risk and subthreshold symptoms. Biological Psychiatry. Cognitive Neuroscience and Neuroimaging, 10(7), 769–780. 10.1016/j.bpsc.2024.06.005. 38909895

[r21] Miller, T. J., McGlashan, T. H., Rosen, J. L., Somjee, L., Markovich, P. J., Stein, K., & Woods, S. W. (2002). Prospective diagnosis of the initial prodrome for schizophrenia based on the structured interview for prodromal syndromes: Preliminary evidence of interrater reliability and predictive validity. The American Journal of Psychiatry, 159(5), 863–865. 10.1176/appi.ajp.159.5.863. 11986145

[r22] Nakadate, K., & Kamata, S. (2022). Severe acute hepatic dysfunction induced by ammonium acetate treatment results in choroid plexus swelling and ventricle enlargement in the brain. International Journal of Molecular Sciences, 23(4). 10.3390/ijms23042010. PMC8879736 35216129

[r23] Nelson, E. A., Kraguljac, N. V., Bashir, A., Cofield, S. S., Maximo, J. O., Armstrong, W., & Lahti, A. C. (2025). A longitudinal study of hippocampal subfield volumes and hippocampal glutamate levels in antipsychotic-naïve first episode psychosis patients. Molecular Psychiatry, 30(5), 2017–2026. 10.1038/s41380-024-02812-1. 39580605 PMC12014507

[r24] Raballo, A., Poletti, M., & Preti, A. (2024). Baseline antipsychotic dose and transition to psychosis in individuals at clinical high risk: A systematic review and meta-analysis. JAMA Psychiatry, 81(7), 727–730. 10.1001/jamapsychiatry.2024.0178. 38506802 PMC10955337

[r25] Reuter, M., Schmansky, N. J., Rosas, H. D., & Fischl, B. (2012). Within-subject template estimation for unbiased longitudinal image analysis. NeuroImage, 61(4), 1402–1418. 10.1016/j.neuroimage.2012.02.084. 22430496 PMC3389460

[r26] Sasabayashi, D., Takayanagi, Y., Takahashi, T., Katagiri, N., Sakuma, A., Obara, C., … Suzuki, M. (2020). Subcortical brain volume abnormalities in individuals with an at-risk mental state. Schizophrenia Bulletin, 46(4), 834–845. 10.1093/schbul/sbaa011. 32162659 PMC7342178

[r27] Seidman, L. J., Faraone, S. V., Goldstein, J. M., Goodman, J. M., Kremen, W. S., Toomey, R., … Tsuang, M. T. (1999). Thalamic and amygdala-hippocampal volume reductions in first-degree relatives of patients with schizophrenia: An MRI-based morphometric analysis. Biological Psychiatry, 46(7), 941–954. 10.1016/s0006-3223(99)00075-x. 10509177

[r28] Sheehan, D. V., Lecrubier, Y., Sheehan, K. H., Amorim, P., Janavs, J., Weiller, E., … Dunbar, G. C. (1998). The Mini-international neuropsychiatric interview (M.I.N.I.): The development and validation of a structured diagnostic psychiatric interview for DSM-IV and ICD-10. The Journal of Clinical Psychiatry, 59(Suppl 20), 22–33 quiz 34–57. 9881538

[r29] Si, S., Bi, A., Yu, Z., See, C., Kelly, S., Ambrogi, S., … Kempton, M. J. (2024). Mapping gray and white matter volume abnormalities in early-onset psychosis: An ENIGMA multicenter voxel-based morphometry study. Molecular Psychiatry, 29(2), 496–504. 10.1038/s41380-023-02343-1. 38195979 PMC11116097

[r30] Sinnecker, T., Ruberte, E., Schädelin, S., Canova, V., Amann, M., Naegelin, Y., … Yaldizli, Ö. (2020). New and enlarging white matter lesions adjacent to the ventricle system and thalamic atrophy are independently associated with lateral ventricular enlargement in multiple sclerosis. Journal of Neurology, 267(1), 192–202. 10.1007/s00415-019-09565-w. 31612322

[r31] Staal, W. G., Hulshoff Pol, H. E., Schnack, H. G., Hoogendoorn, M. L., Jellema, K., & Kahn, R. S. (2000). Structural brain abnormalities in patients with schizophrenia and their healthy siblings. The American Journal of Psychiatry, 157(3), 416–421. 10.1176/appi.ajp.157.3.416. 10698818

[r32] Tourjman, V., Kouassi, É., Koué, M., Rocchetti, M., Fortin-Fournier, S., Fusar-Poli, P., & Potvin, S. (2013). Antipsychotics’ effects on blood levels of cytokines in schizophrenia: A meta-analysis. Schizophrenia Research, 151(1–3), 43–47. 10.1016/j.schres.2013.10.011. 24200418

[r33] Vernon, A. C., Natesan, S., Modo, M., & Kapur, S. (2011). Effect of chronic antipsychotic treatment on brain structure: A serial magnetic resonance imaging study with ex vivo and postmortem confirmation. Biological Psychiatry, 69(10), 936–944. 10.1016/j.biopsych.2010.11.010. 21195390

[r34] Vissink, C. E., Winter-van Rossum, I., Cannon, T. D., Fusar-Poli, P., Kahn, R. S., & Bossong, M. G. (2022). Structural brain volumes of individuals at clinical high risk for psychosis: A meta-analysis. Biological Psychiatry Global Open Science, 2(2), 147–152. 10.1016/j.bpsgos.2021.09.002. 36325161 PMC9616363

[r35] Zeng, J., Zhang, W., Wu, G., Wang, X., Shah, C., Li, S., … Gong, Q. (2022). Effects of antipsychotic medications and illness duration on brain features that distinguish schizophrenia patients. Schizophrenia Bulletin, 48(6), 1354–1362. 10.1093/schbul/sbac094. 35925035 PMC9673268

[r36] Zhang, T., Li, H., Woodberry, K. A., Seidman, L. J., Zheng, L., Li, H., … Wang, J. (2014). Prodromal psychosis detection in a counseling center population in China: An epidemiological and clinical study. Schizophrenia Research, 152(2–3), 391–399. 10.1016/j.schres.2013.11.039. 24387999 PMC4441955

[r37] Zhang, T., Raballo, A., Zeng, J., Gan, R., Wu, G., Wei, Y., … Wang, J. (2022). Antipsychotic prescription, assumption and conversion to psychosis: Resolving missing clinical links to optimize prevention through precision. Schizophrenia (Heidelb), 8(1), 48. 10.1038/s41537-022-00254-8. 35853891 PMC9261109

[r38] Zhang, T., Xu, L., Tang, X., Wei, Y., Hu, Q., Hu, Y., … Wang, J. (2020). Real-world effectiveness of antipsychotic treatment in psychosis prevention in a 3-year cohort of 517 individuals at clinical high risk from the SHARP (ShangHai at risk for psychosis). The Australian and New Zealand Journal of Psychiatry, 54(7), 696–706. 10.1177/0004867420917449. 32436725

[r39] Zhang, T., Xu, L., Wei, Y., Tang, X., Hu, Y., Cui, H., … Wang, J. (2021). When to initiate antipsychotic treatment for psychotic symptoms: At the premorbid phase or first episode of psychosis? The Australian and New Zealand Journal of Psychiatry, 55(3), 314–323. 10.1177/0004867420969810. 33143440

[r40] Zhang, W., Qiu, C., & Lui, S. (2025). Imaging biomarker studies of antipsychotic-naïve first-episode schizophrenia in China: Progress and future directions. Schizophrenia Bulletin, 51(2), 379–391. 10.1093/schbul/sbaf002. 39841545 PMC11908865

[r41] Zhao, T., Chen, A., Dai, D., Li, Z., Feng, E., Gao, X. F., & Xiong, L. (2025). Down-regulation of gastrin-releasing peptide in medial orbitofrontal cortex to nucleus accumbens projections contributes to allodynia and negative affect. Science Advances, 11(50), eadz1614. 10.1126/sciadv.adz1614. 41370385 PMC12694044

[r42] Zhao, Y., Zhang, Q., Shah, C., Li, Q., Sweeney, J. A., Li, F., & Gong, Q. (2022). Cortical thickness abnormalities at different stages of the illness course in schizophrenia: A systematic review and meta-analysis. JAMA Psychiatry, 79(6), 560–570. 10.1001/jamapsychiatry.2022.0799. 35476125 PMC9047772

[r43] Zheng, W., Xu, L., Zhang, D., Su, W., Wei, Y., Cui, H., … Wang, J. (2025). The effects of antipsychotics on two month cortical thickness and two year clinical outcomes among populations at clinical high risk for psychosis. Schizophrenia Bulletin. 10.1093/schbul/sbaf111. PMC13391646 40801809

