# Supplementary material for: Distinct brain volume abnormalities in clinical high-risk individuals: pre- and post-antipsychotic treatment
Source: Psychol Med. 2026 Mar 24;56:e74. doi: 10.1017/S0033291726103250 (PMC13079220; doi:10.1017/S0033291726103250)
Supplement: Zheng et al. supplementary material [file S0033291726103250sup001.docx]

### **Supplementary Material**

**Table S1. Detailed Desikan-Killiany Labels Included in Each Cortical Region Used in the Study**

| **Lobes** | **Abbreviation** | **Desikan-Killiany ROIs** |
| --- | --- | --- |
| Orbitofrontal Cortex | OFC | Lateral Orbitofrontal |
|  |  | Medial Orbitofrontal |
|  |  | Pars Orbitalis |
| Lateral Prefrontal Cortex | LPFC | Frontal Pole |
|  |  | Pars Opercularis |
|  |  | Pars Triangularis |
|  |  | Rostral Middle Frontal |
| Medial Prefrontal Cortex | MPFC | Caudal Anterior Cingulate |
|  |  | Rostral Anterior Cingulate |
|  |  | Superior Frontal |
| Lateral Temporal Cortex | LTC | Bankssts |
|  |  | Inferior Temporal |
|  |  | Middle Temporal |
|  |  | Superior Temporal |
|  |  | Temporal Pole |
|  |  | Transverse Temporal |
| Medial Temporal Cortex | MTC | Entorhinal |
|  |  | Fusiform |
|  |  | Parahippocampal |
| Somatomotor Cortex | SMC | Caudal Middle Frontal |
|  |  | Paracentral |
|  |  | Postcentral |
|  |  | Precentral |
| Parietal Cortex | PC | Inferior Parietal |
|  |  | Isthmus Cingulate |
|  |  | Posterior Cingulate |
|  |  | Precuneus |
|  |  | Superior Parietal |
|  |  | Supramarginal |
| Occipital Cortex | OCC | Cuneus |
|  |  | Lateral Occipital |
|  |  | Lingual |
|  |  | Pericalcarine |

**Table S2. Group Comparisons Between CHR and HC in All ROIs at Baseline**

| Brain Region | β | SE | t | p | FDR-p |
| --- | --- | --- | --- | --- | --- |
| X3rd ventricle | 164.68 | 52.39 | 3.14 | 0.002 | 0.017* |
| Lateral ventricle | 2277.97 | 942.00 | 2.42 | 0.016 | 0.074 |
| Inferior lateral ventricle | 181.55 | 51.72 | 3.51 | <0.001 | 0.010* |
| Thalamus | -149.65 | 144.95 | -1.03 | 0.303 | 0.420 |
| Caudate | 96.94 | 111.51 | 0.87 | 0.386 | 0.496 |
| Putamen | 178.77 | 135.26 | 1.32 | 0.188 | 0.375 |
| Pallidum | 103.80 | 54.71 | 1.90 | 0.059 | 0.178 |
| Hippocampus | -29.45 | 105.41 | -0.28 | 0.780 | 0.826 |
| Amygdala | 46.14 | 41.66 | 1.11 | 0.269 | 0.404 |
| Accumbens | -23.35 | 19.72 | -1.18 | 0.238 | 0.404 |
| OFC | 257.64 | 422.34 | 0.61 | 0.543 | 0.610 |
| LPFC | 1793.21 | 791.21 | 2.27 | 0.024 | 0.088 |
| MPFC | 1640.55 | 663.36 | 2.47 | 0.014 | 0.074 |
| LTC | 1436.18 | 1261.25 | 1.14 | 0.256 | 0.404 |
| MTC | 95.85 | 463.91 | 0.21 | 0.837 | 0.837 |
| SMC | 1352.70 | 869.71 | 1.56 | 0.121 | 0.273 |
| PC | 964.48 | 1402.81 | 0.68 | 0.493 | 0.591 |
| OCC | 1346.70 | 796.40 | 1.69 | 0.092 | 0.237 |

**Table S3. Correlations Between ROI Volumes and Clinical Variables at Baseline**

| Clinical Variable | Brain Region | r | p | FDR-p |
| --- | --- | --- | --- | --- |
| SOPS Total | X3rd ventricle | 0.26 | 0.001 | 0.011* |
|  | Lateral ventricle | 0.27 | <0.001 | 0.009** |
|  | Inferior lateral ventricle | 0.26 | 0.001 | 0.011* |
|  | Thalamus | -0.16 | 0.050 | 0.128 |
|  | Caudate | -0.02 | 0.823 | 0.887 |
|  | Putamen | 0.01 | 0.870 | 0.887 |
|  | Pallidum | -0.05 | 0.585 | 0.675 |
|  | Hippocampus | -0.21 | 0.011 | 0.041* |
|  | Amygdala | -0.26 | 0.002 | 0.011* |
|  | Accumbens | -0.13 | 0.115 | 0.213 |
|  | OFC | -0.13 | 0.118 | 0.213 |
|  | LPFC | -0.04 | 0.657 | 0.740 |
|  | MPFC | -0.06 | 0.470 | 0.611 |
|  | LTC | -0.20 | 0.014 | 0.052 |
|  | MTC | -0.17 | 0.043 | 0.122 |
|  | SMC | 0.02 | 0.855 | 0.887 |
|  | PC | -0.17 | 0.045 | 0.122 |
|  | OCC | -0.13 | 0.113 | 0.213 |
| GAF Current | X3rd ventricle | -0.24 | 0.003 | 0.016* |
|  | Lateral ventricle | -0.27 | <0.001 | 0.009** |
|  | Inferior lateral ventricle | -0.23 | 0.006 | 0.028* |
|  | Thalamus | 0.11 | 0.172 | 0.282 |
|  | Caudate | -0.06 | 0.485 | 0.611 |
|  | Putamen | -0.10 | 0.234 | 0.361 |
|  | Pallidum | -0.05 | 0.588 | 0.675 |
|  | Hippocampus | 0.30 | <0.001 | 0.009** |
|  | Amygdala | 0.25 | 0.002 | 0.012* |
|  | Accumbens | 0.15 | 0.069 | 0.155 |
|  | OFC | 0.16 | 0.055 | 0.136 |
|  | LPFC | 0.01 | 0.871 | 0.887 |
|  | MPFC | 0.11 | 0.173 | 0.282 |
|  | LTC | 0.16 | 0.060 | 0.140 |
|  | MTC | 0.19 | 0.023 | 0.073 |
|  | SMC | 0.02 | 0.809 | 0.887 |
|  | PC | 0.06 | 0.450 | 0.608 |
|  | OCC | 0.07 | 0.380 | 0.555 |
| GAF Drop Rate | X3rd ventricle | 0.22 | 0.008 | 0.035* |
|  | Lateral ventricle | 0.29 | <0.001 | 0.009** |
|  | Inferior lateral ventricle | 0.25 | 0.002 | 0.012* |
|  | Thalamus | -0.13 | 0.132 | 0.229 |
|  | Caudate | 0.06 | 0.495 | 0.611 |
|  | Putamen | 0.09 | 0.266 | 0.398 |
|  | Pallidum | 0.06 | 0.498 | 0.611 |
|  | Hippocampus | -0.28 | <0.001 | 0.009** |
|  | Amygdala | -0.18 | 0.029 | 0.088 |
|  | Accumbens | -0.14 | 0.097 | 0.201 |
|  | OFC | -0.13 | 0.111 | 0.213 |
|  | LPFC | -0.07 | 0.418 | 0.579 |
|  | MPFC | -0.07 | 0.402 | 0.571 |
|  | LTC | -0.14 | 0.097 | 0.201 |
|  | MTC | -0.19 | 0.020 | 0.067 |
|  | SMC | 0.002 | 0.980 | 0.980 |
|  | PC | -0.05 | 0.552 | 0.663 |
|  | OCC | -0.10 | 0.221 | 0.350 |

**Table S4. Linear Mixed-Effect Model Results for Subgroup Analysis of ROIs with Significant Time*Group Interactions Between CHR and HC**

|  | X3rd Ventricle | Lateral Ventricle | Inferior Lateral Ventricle | Accumbens | OFC | LPFC | MPFC | LTC | MTC | SMC | OCC |
| --- | --- | --- | --- | --- | --- | --- | --- | --- | --- | --- | --- |
| Group: LOW vs HIGH vs HC | | | | | | | | | | | |
| (BL-M2) x (LOW-HC) | 31.60 (11.68)** | 456.03 (142.41)** | 54.44 (18.10)** | -25.88 (12.88)* | -108.95 (169.65) | -675.48 (326.38)* | -973.70 (370.75)** | -1117.48 (458.25)* | -197.43 (159.86) | -1085.66 (466.58)* | -459.24 (302.57) |
| (BL-M2) x (HIGH-HC) | 40.74 (12.24)** | 540.27 (149.21)*** | 65.55 (18.97)*** | -37.60 (13.51)** | -766.17 (177.82)*** | -1531.80 (342.11)*** | -1583.62 (388.76)*** | -2323.01 (480.27)*** | -589.93 (167.54)*** | -1472.05 (489.26)** | -1041.25 (317.13)** |
| Post hoc: HC (BL-M2) | 5.24 (15.41) | -90.68 (188.27) | 26.55 (23.78) | 3.22 (16.61) | -115.53 (222.56) | -363.52 (427.91) | -338.14 (481.95) | -894.08 (602.28) | -56.48 (210.19) | -466.87 (606.43) | 38.30 (396.99) |
| Post hoc: LOW (BL-M2) | -26.36 (13.20)* | -546.70 (161.27)*** | -27.88 (20.38) | 29.10 (14.26)* | -6.58 (190.79) | 311.96 (366.85) | 635.57 (413.52) | 223.41 (516.22) | 140.95 (180.15) | 618.79 (520.34) | 497.54 (340.32) |
| Post hoc: HIGH (BL-M2) | -35.49 (14.73)* | -630.95 (179.85)*** | -38.99 (22.74) | 40.82 (15.91)* | 650.63 (212.79)** | 1168.27 (409.16)** | 1245.48 (461.28)** | 1428.94 (575.74)* | 533.45 (200.92)** | 1005.19 (580.44) | 1079.55 (379.57)** |
| Group: LOW_R vs LOW_NR vs HIGH_R vs HIGH_NR vs HC | | | | | | | | | | | |
| (BL-M2) x (LOW_R-HC) | 29.80 (15.53) | 454.27 (187.29)* | 74.92 (23.80)** | -36.96 (17.20)* | -175.07 (224.16) | -996.40 (420.02)* | -1168.70 (474.61)* | -964.96 (610.41) | -96.18 (212.92) | -1169.49 (601.93) | -185.94 (398.08) |
| (BL-M2) x (LOW_NR-HC) | 35.80 (13.50)** | 484.11 (162.78)** | 46.06 (20.68)* | -23.46 (14.94) | -65.72 (194.80) | -549.41 (365.00) | -862.54 (412.39)* | -1309.70 (530.47)* | -276.40 (185.03) | -980.92 (523.02) | -620.63 (345.94) |
| (BL-M2) x (HIGH_R-HC) | 50.14 (15.54)** | 819.19 (187.48)*** | 91.76 (23.83)*** | -40.87 (17.23)* | -763.27 (224.44)*** | -2005.39 (420.55)*** | -2453.76 (475.35)*** | -3034.20 (611.16)*** | -770.76 (213.17)*** | -2538.86 (602.89)*** | -1557.92 (398.59)*** |
| (BL-M2) x (HIGH_NR-HC) | 38.23 (15.32)* | 339.17 (184.75) | 45.10 (23.48) | -38.31 (16.98)* | -805.94 (221.18)*** | -1324.15 (414.44)** | -1086.22 (468.46)* | -1839.78 (602.27)** | -463.82 (210.07)* | -823.20 (594.14) | -779.02 (392.80)* |
| Post hoc: HC (BL-M2) | 7.58 (15.74) | -56.58 (190.19) | 28.63 (24.01) | 1.28 (17.03) | -118.33 (225.82) | -435.54 (423.17) | -390.28 (474.48) | -986.41 (616.03) | -68.39 (214.99) | -479.95 (601.41) | 14.04 (401.19) |
| Post hoc: LOW_R (BL-M2) | -22.22 (16.75) | -510.85 (202.22)* | -46.29 (25.61) | 38.25 (18.32)* | 56.74 (241.00) | 560.86 (451.59) | 778.42 (508.21) | -21.45 (656.89) | 27.79 (229.19) | 689.54 (644.35) | 199.98 (428.08) |
| Post hoc: LOW_NR (BL-M2) | -28.22 (14.83) | -540.69 (179.12)** | -17.43 (22.66) | 24.75 (16.16) | -52.61 (213.20) | 113.87 (399.51) | 472.27 (449.04) | 323.29 (581.28) | 208.02 (202.83) | 500.97 (569.28) | 634.67 (378.73) |
| Post hoc: HIGH_R (BL-M2) | -42.56 (17.87)* | -875.78 (215.84)*** | -63.14 (27.32)* | 42.15 (19.52)* | 644.94 (257.07)* | 1569.85 (481.72)** | 2063.49 (541.79)*** | 2047.79 (700.80)** | 702.37 (244.53)** | 2058.91 (686.89)** | 1571.96 (456.65)*** |
| Post hoc: HIGH_NR (BL-M2) | -30.66 (17.87) | -395.76 (215.83) | -16.47 (27.31) | 39.60 (19.50)* | 687.61 (257.00)** | 888.61 (481.58) | 695.94 (541.50) | 853.36 (700.63) | 395.43 (244.47) | 343.24 (686.52) | 793.06 (456.52) |

Note: The variables for all brain regions are presented in the form of β (SE). β, estimated effect; SE, Standard Error. *Significant at p<0.05, **Significant at p<0.01, ***Significant at p<0.001.


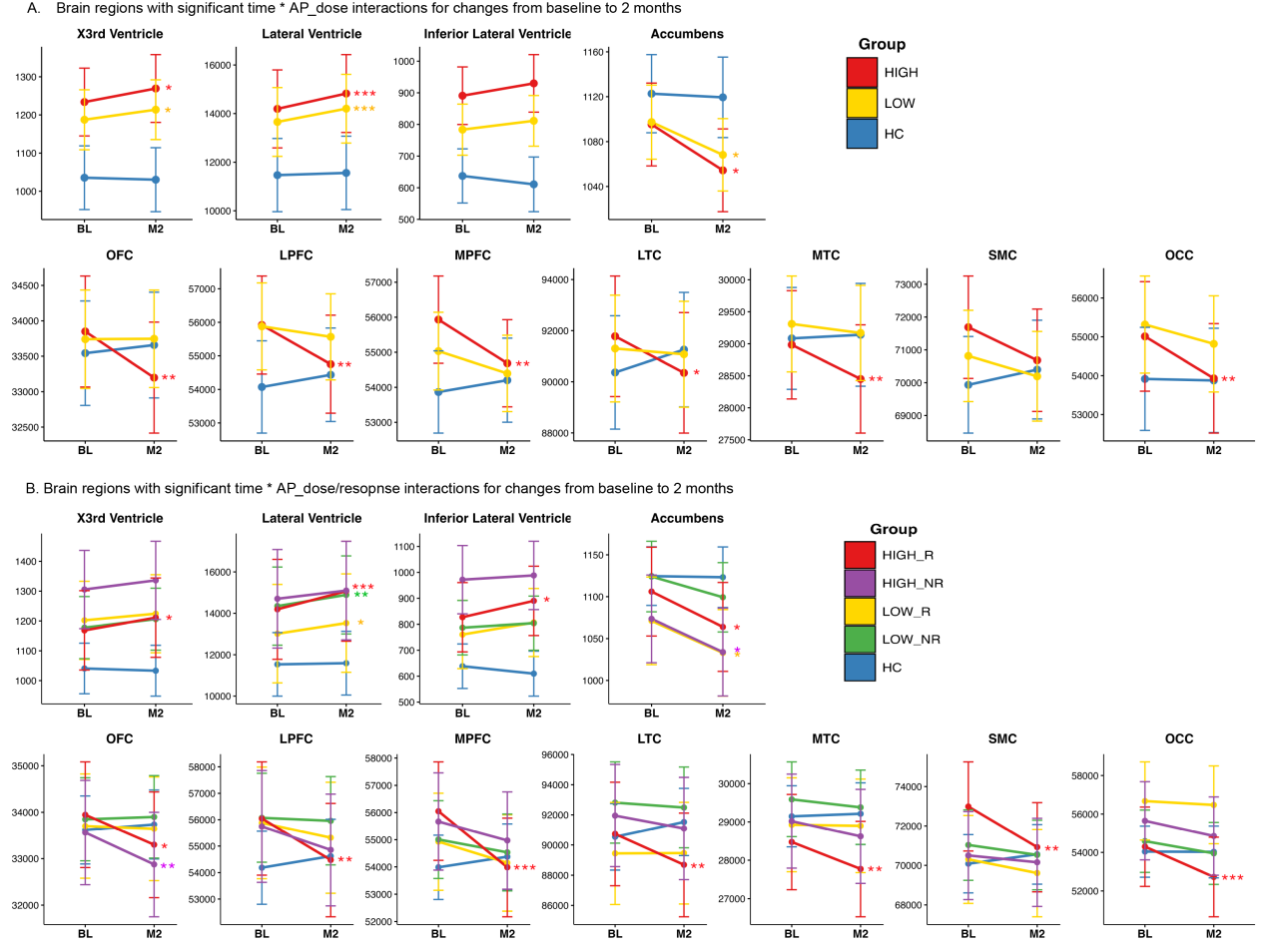


**Figure S1. Marginal Mean Plots of ROIs with Significant Group*Time Interactions Across AP-dose/response Subgroups.** Both LOW and HIGH groups showed greater volumetric expansion in the 3rd, lateral, and inferior lateral ventricles, and faster volume reduction in the accumbens compared to HCs. In cortical ROIs, the HIGH group showed more significant cortical volume reductions than the LOW group compared with HC (A). The 3rd ventricle expanded faster in the LOW_NR, HIGH_R, and HIGH_NR groups compared to HC, with greater expansion in the lateral and inferior lateral ventricles in LOW_R, LOW_NR, and HIGH_R. The accumbens showed faster decline in LOW_R, HIGH_R, and HIGH_NR groups. For cortical regions, OFC showed similar reductions in HIGH_R and HIGH_NR groups, while the most significant changes in other regions (LPFC, MPFC, LTC, MTC, SMC, OCC) were found in the HIGH_R group (B). Simple effects: *p < 0.05, **p < 0.01, ***p < 0.001.
